# Conjugative transfer of ICE*Sde*3396 between three β-hemolytic streptococcal species

**DOI:** 10.1186/1756-0500-7-521

**Published:** 2014-08-12

**Authors:** Danielle J Smyth, Josephine Shera, Michelle J Bauer, Ainslie Cameron, Celia L McNeilly, Kadaba S Sriprakash, David J McMillan

**Affiliations:** Bacterial Pathogenesis Laboratory, QIMR Berghofer Medical Research Institute, 300 Herston Rd, Herston, QLD 4006 Australia; Inflammation and Healing Research Cluster, School of Health and Sport Sciences, University of the Sunshine Coast, Maroochydore, Queensland, 4558 Australia; Institute of Immunology and Infection Research, University of Edinburgh, Ashworth Laboratories, West Mains Road, Edinburgh, EH9 3JT UK

**Keywords:** Integrative conjugative element, Conjugation, *Streptococcus dysgalactiae* subsp. *equisimilis*, *Streptococcus pyogenes*, Streptococcus *agalactiae*

## Abstract

**Background:**

Integrative conjugative elements (ICEs) are mobile genetic elements (MGEs) that possess all genes necessary for excision, transfer and integration into recipient genome. They also carry accessory genes that impart new phenotypic features to recipient strains. ICEs therefore play an important role in genomic plasticity and population structure. We previously characterised ICE*Sde*3396, the first ICE identified in the β-hemolytic *Streptococcus dysgalactiae* subsp *equisimilis* (SDSE) and demonstrated its transfer to single isolates of *Streptococcus pyogenes* (group A streptococcus, GAS) and *Streptococcus agalactiae* (group B streptococcus, GBS). While molecular studies found the ICE in multiple SDSE and GBS isolates, it was absent in all GAS isolates examined.

**Results:**

Here we demonstrate that ICE*Sde*3396:km is transferable from SDSE to multiple SDSE, GAS and GBS isolates. However not all strains of these species were successful recipients under the same growth conditions. To address the role that host factors may have in conjugation we also undertook conjugation experiments in the presence of A549 epithelial cells and DMEM. While Horizontal Gene Transfer (HGT) occurred, conjugation efficiencies were no greater than when similar experiments were conducted in DMEM. Additionally transfer to GAS NS235 was successful in the presence of DMEM but not in Todd Hewitt Broth suggesting that nutritional factors may also influence HGT. The GAS and GBS transconjugants produced in this study are also able to act as donors of the ICE.

**Conclusion:**

We conclude that ICEs are major sources of interspecies HGT between β-hemolytic streptococci, and by introducing accessory genes imparting novel phenotypic characteristics, have the potential to alter the population structure of these species.

## Background

*Streptococcus pyogenes* (group A streptococcus; GAS), *S. agalactiae* (group B streptococcus; GBS) and *S. dysgalactiae* subsp. *equisimilis* (SDSE) are related Gram-positive β-hemolytic bacteria that can cause a number of potentially fatal diseases in humans [1–4]. Two of these species, GAS and GBS are major human pathogens. The third, SDSE, generally considered an opportunistic pathogen, causes a similar spectrum of diseases to that of GAS, and is more closely related to this organism than to GBS.

Genetic and genomic studies have revealed an extensive history of intra-species horizontal gene transfer (HGT). Although genetic evidence for interspecies HGT is also present, the mechanisms of this HGT have yet to be full elucidated. Indeed, comparative genomics of GAS and GBS, the two major pathogens, have revealed the importance of HGT and Mobile Genetic Elements (MGEs) to the genomic plasticity of these two species [5–11]. These studies also revealed that different classes of MGEs, namely Integrative Conjugative Elements (ICEs) and bacteriophages, contribute to the genomic plasticity to the two species at different frequencies. Whereas most GAS isolates are polylysogenic, phages are less prevalent in the GBS population. No evidence for HGT involving bacteriophage between the two species has been reported. In contrast, ICEs are prominent in GBS [11, 12], but less so in the GAS population. Unlike bacteriophages, evidence for the transfer of ICEs between GBS and GAS has also been reported [6]. Our knowledge of the genetics and population structure of SDSE is limited when compared to the two major pathogens of the genus. The SDSE strain GGS124 [13] contains two prophages, whilst D166b [9] contains none. Exotoxin genes, which are used as surrogate markers for the presence of bacteriophage in GAS, are also not found in either SDSE genome suggesting that interspecies transfer of exotoxin- bearing bacteriophages between the two species is absent or rare [14].

We have previously reported the identification and characterisation of the first ICE in SDSE. ICE*Sde*3396 encodes 66 open reading frames, and possesses the genes encoding machinery necessary for excision, transfer and integration into the recipient chromosome [15]. The ICE also possesses an internal 18 kb region (Region 2) that harbours several functional accessory genes cassettes. The closest orthologues of several of the genes are found in other bacterial genera. Using probes that target three regions of the ICE we also demonstrated that approximately 50% of SDSE and GBS isolates contain the ICE or variants thereof. In contrast, no ICE was present in any of the GAS isolates examined. However we demonstrated transfer of the ICE to one GAS strain under laboratory conditions. In the current study we demonstrate that ICE*Sde*3396:km is transferable from both SDSE and GBS to GAS, and that the ICE is stably integrated into the chromosome of all three species. The transconjugant GAS and GBS are also able to act as donor of the ICE. Our data support a model where ICEs are the major MGE involved in inter-species dissemination of new genes throughout the β-hemolytic streptococcal population.

## Results

### Time course of ICE*Sde*3396:km transfer

**Figure 1:**
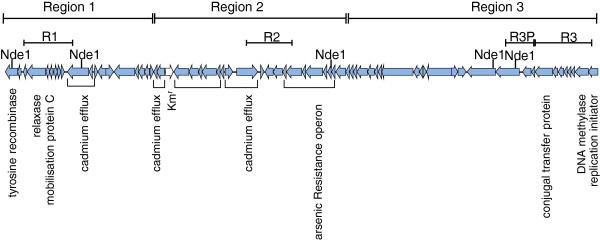
**Schematic diagram of ICE** ***Sde*** **3396:km (not to scale).** The three regions (Region 1, Region 2 and Region 3) of the ICE, and regions targeted by PCR to determine the presence of these regions (i.e. R1, R2 and R3) are shown at the top of figure. R3P depicts the location of the probe used in Southern hybridisation. The location of *Nde*I sites and kanamycin resistance gene (unfilled arrow) are also shown. Genes involved in mobilisation, integration into recipient chromosomes, and those involved in conferring resistance to arsenate and cadmium are shown.

To analyse the kinetics of transfer to different streptococcal species, we developed a microfuge based method that enabled sampling of conjugation suspensions at different time points (10 min - 3 h). The assay also utilised SDSE NS3396 ICE*Sde*3396:km as the donor strain (Figure 1). The ICE in this strain has been modified by the insertion of a kanamycin resistance gene that was used as the ICE selectable marker in all experiments. Using this model we were able to transfer the ICE to SDSE GGS10str within as little as 30 min in three independent experiments, and in as little as 10 minutes in one instance (Table 1). Transfer of the ICE to GBS RBH05str was also observed under these conditions, but occurred later than transfer to SDSE, with transconjugants infrequently recovered prior to 1 hour post-incubation. Conjugation efficiencies between SDSE and GBS were also less than those observed with SDSE-SDSE transfers. Under the experimental conditions used here we were unable to demonstrate transfer to GAS NS235str at any time point. To further investigate the transfer of the ICE to GAS NS235str, mating experiments were repeated multiple times and with extended incubation periods. In all instances, we were unable to transfer the ICE to this GAS strain, whereas transfer of the ICE to SDSE GGS10str, used as control for the experiment, occurred on all occasions. Together this data suggests that specific factors may inhibit the transfer of ICEs to GAS but not interfere with transfer to SDSE or GGS.

**Table 1:** **Conjugation frequency of ICE** ***Sde*** **3396:km using SDSE NS3396:km as donor strain**

Recipient	Time (min)				
	10	30	60	120	180
SDSE GGS10str	2.4 × 10^-7^	4.3 × 10^-7^	7.6 × 10^-7^	4.1 × 10^-6^	6.3 × 10^-6^
GBS RBH05str	0	0	7.5 × 10^-8^	0	7.9 × 10^-8^
GAS NS235str	0	0	0	0	0

### ICESde3396:km is transferable to SDSE, GBS and GAS in the presence of eukaryotic cells

The inability to transfer the ICE to GAS in this model is consistent with our epidemiological data [15] demonstrating the absence of ICE*Sde*3396 in the GAS isolates, and suggests that genetic or environmental factors may inhibit the transfer or maintenance of ICE*Sde*3396 in GAS. It has been reported that growth of GAS in the presence of pharyngeal cells results in bacteriophage induction [16], theoretically increasing the incidence of HGT. To investigate whether co-culturing also promote HGT of ICEs, we next conducted mating experiments in the presence of A549 epithelial cells. Transfer of the ICE to GGS10str was observed in the presence of A549 cells in 2 of 3 independent experiments (Table 2). Transfer was also observed in 2 of 3 independent experiments when spent medium was used. Successful transfer in all experiments (6/6) was observed using fresh DMEM. The mean conjugation efficiencies for all these experiments were less than that observed when experiments were performed using the THB/microfuge model. When GBS RBH05str was used as the recipient, successful transfer was also observed in 2 of 3 experiments in the presence of A549 cells, 3/3 in the presence of spent media, and all 6 experiments when using DMEM. Using this model we were also successful in transferring the ICE to GAS NS235str. In the presence of fresh DMEM transfer was observed in 3/6 experiments. In the presence of A549 cells and spent media transfer was observed in 2 of 3 experiments respectively. Although the number of successful independent transfer experiments was less than when SDSE and GBS were used as donors, the conjugation frequencies were not dissimilar, and there was no significant difference between any of the conjugation frequencies for any treatment group. To assess recipient strain specificity in this model additional conjugation experiments, using multiple recipient strains, were performed. Successful transfer of the ICE occurred between SDSE NS3396 and four of four additional SDSE recipient strains, three of five additional GAS strains and five of eight GBS strains (Table 3). Taken together our results demonstrate that the ICE is transferable to all three streptococcal species, but not all strains of a particular species in a given growth condition.

**Table 2:** **Transfer of ICE** ***Sde*** **3396 from SDSE NS3396:km**

Recipient	Growth conditions	Successful conjugation/total experiments	Average conjugation frequency
SDSE GGS10str	DMEM	6/6	1.8 × 10^-8^
	Spent medium	2/3	5.9 × 10^-9^
	A549 cells	2/3	3.8 × 10^-8^
GAS NS235str	DMEM	3/6	4.0 × 10^-9^
	Spent Medium	2/3	1.7 × 10^-9^
	A549 cells	2/3	2.0 × 10^-9^
GBS RBH05str	DMEM	6/6	9.5 × 10^-9^
	Spent medium	3/3	7.6 × 10^-10^
	A549 cells	2/3	3.8 × 10^-8^

**Table 3:** **Transfer of ICE** ***Sde*** **3396 to multiple isolates of SDSE, GAS and GBS**

Recipient species	Recipient strain	emm- type/serotype	Transconjugants
SDSE	G120	stg4831	yes
	MD128	stg93464	yes
	NS1121	stg4831	yes
	NS383	New type	yes
GAS	NS1185	n.d.^a^	no
	NS20	emm75.1	yes
	NS344	emm1	yes
	NS351	emm58	yes
	NS672	n.d.	no
GBS	P36PS	IV	no
	RBH04	Ia/V	yes
	RBH06	II	yes
	RBH08	Ia	no
	RBH09	V	yes
	RBH10	V	yes
	RBH11	III	yes
	RBH14	Ib	no

^a^n.d. not determined.

### GAS and GBS transconjugants are donors of ICE*Sde*3396:km

**Figure 2:**
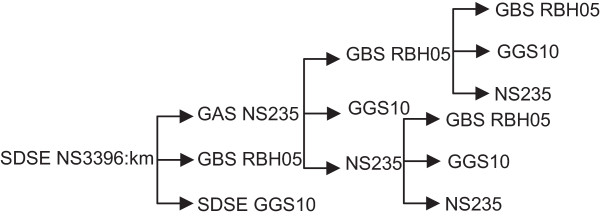
**ICE** ***Sde*** **3396 is transferable from SDSE, GAS and GBS.** Arrows represent direction of transfer of the ICE into recipient strains. All three species are capable of acting as donors and recipients of the ICE. Transconjugants were initially identified on the basis of double antibiotic resistance phenotype. Transfer of the full ICE was confirmed by PCR amplification of DNA from Regions 1, 2 and 3 (data not shown).

To determine whether GAS and GBS transconjugants were also capable of acting as donors of ICE*Sde*3396:km, GAS NS235str::ICE*Sde*3396:km and GBS RBH05str::ICE*Sde*3396:km, transconjugants produced in earlier experiments were used as donors in a new round of conjugations. As these donor strains were also streptomycin-resistant, the inherent bacitracin resistance present in SDSE and GBS was used as the selectable marker for these recipients. To monitor transfer of the ICE into GAS, a spectinomycin resistant GAS NS235 was generated. For both donors, we were able to transfer the ICE to recipients of all three species, as determined on the basis of a double antibiotic resistance phenotype. Figure 2 summarises the path of transfer of the ICE through serial conjugation experiments conducted in the study. The presence of full length ICE in each transconjugants was confirmed by PCR of the R1, R2 and R3 regions (data not shown).

### ICE*Sde*3396:km is stably integrated into the chromosome

**Figure 3:**
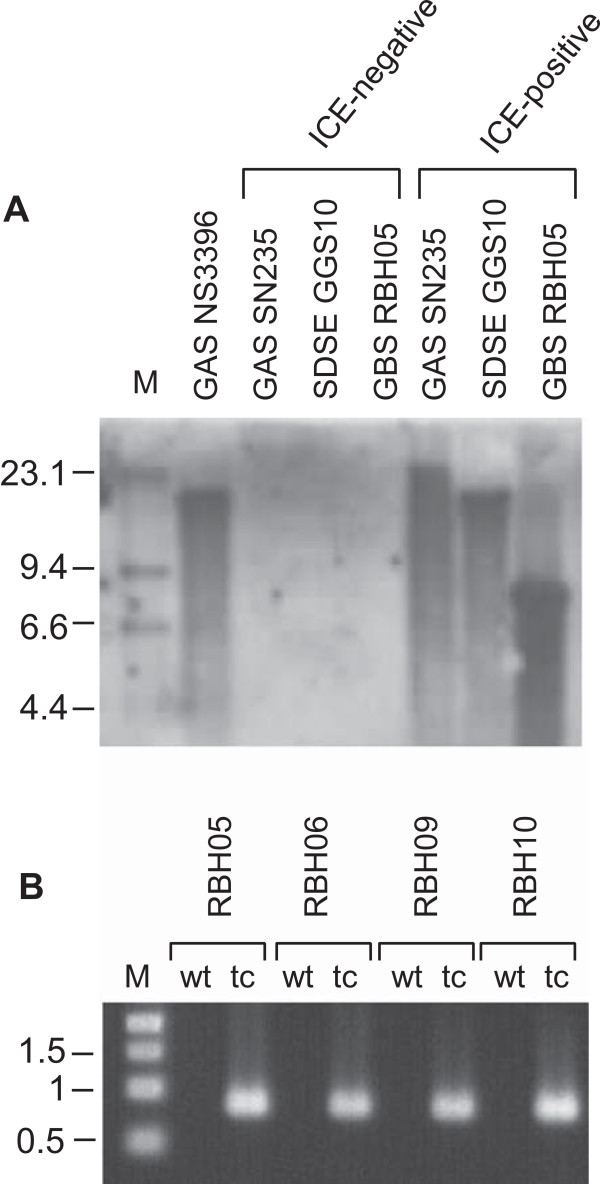
**ICE** ***Sde*** **3396:km is chromosomally integrated in SDSE, GBS and GAS. (A)** Southern hydridisation of *Nde*I restricted streptococcal chromosomal DNA probed with R3P. The presence of reactive bands greater than 8.0 kb in GAS and SDSE transconjugants is indicative of chromosomal integration of the ICE. **(B)** PCR amplification of the terminal region of ICESde3396:km and chromosomally encoded *rpl*L gene from ICE-negative wild-type GBS (wt), and corresponding ICE-positive transconjugants (tc) from group B streptococcus, demonstrating chromosomal integration of the ICE in transconjugants.

ICEs can exist as chromosomal integrants or as autonomously replicating episomal elements [17]. In the absence of selection pressure the latter may result in a more unstable acquisition that may be lost during replication. To determine if the status of ICE*Sde*3396 differed between GAS, GBS and SDSE, Southern blots were performed on *Nde*I digested DNA recovered from transconjugants of each species. The R3P probe used for these assays distinguishes between episomal and integrated forms of the ICE. In its episomal form, R3P reacts with a 8.0 kb fragment flanked by *Nde*I sequences present in the ICE, and which span the *att* site. When integrated into the chromosome, one NdeI site of the reactive band is present in the ICE, and other is present in the chromosome. The size of the reactive band when the ICE was integrated into the chromosome is therefore dependent on the location of the chromosomal *Nde*I site adjoining the attachment site (*attB*). For all GAS and SDSE transconjugants examined, the reactive band observed was greater than 8.0 kb, indicating that the ICE was chromosomally integrated (Figure 3A). In contrast, the reactive band in GBS RBH06 transconjugants was 8.0 kb. Subsequent analysis of published GBS genomes identified an *Nde*I site situated in the chromosome such that a reactive band would also be 8.0 kb in size when probed with R3P. PCR analysis using primers that span the *attB* site in multiple GBS transconjugants subsequently demonstrated the ICE was integrated into the chromosome in this species (Figure 3B).

## Discussion

Within GAS MGEs are reported contribute to the pathogenesis of specific strains by enabling the acquisition of virulence genes from other GAS isolates [18, 19], or other beta-hemolytic streptococci [6]. Thus the mechanisms that promote or inhibit HGT within this group of bacteria are of particular interest. Given the lack of evidence of inter-species bacteriophage transfer between different β-hemolytic streptococci, ICE mediated HGT is likely to be the major transfer mechanism between these species. This is the first study demonstrating direct transfer of ICEs from GBS to GAS, as well as demonstrating that ICEs can be transferred between GAS, SDSE and GBS. Thus the three major β-hemolytic species colonising and causing disease in humans constitute an open group of micro-organisms in which bidirectional ICE-mediated HGT can occur.

While our success in transferring the ICE to multiple GAS isolates appears contradictory to our previous epidemiological results, our inability to transfer the ICE to all recipients suggest that strain-specific factors may play a role in acquisition of the ICE. While none of the GAS recipient strains used in this study possessed ICE*Sde*3396 (data not shown), we cannot exclude the possibility that other MGEs are present in the *rpl*L integration site, a known hotspot for ICE integration [20]. Alternate HGT inhibitory mechanisms such as active ICE exclusion [21], or prophage derived inhibitory factors [22] have also been reported in other bacteria. Given the polylysogenic nature of GAS, it is tempting to suggest such a mechanism is at least partly responsible for the reduction in ICE mediated HGT in this study and would be consistent with the failure to detect ICE*Sde*3396 in the natural GAS population [15].

Although we successfully transferred the ICE in the presence of A459 cells, conjugation frequency was no greater than that observed when fresh or spent DMEM, suggesting that the eukaryotic cells play no part in promoting ICE mediated HGT. Interestingly in the case of GAS NS235, we were able to demonstrate transfer from SDSE GGS 3396:km in the presence of DMEM, but not THB, providing some evidence that environmental factors influence ICE transfer, at least for this strain. Of note when we replaced the Todd Hewitt broth with DMEM in the microfuge model, transfer of the ICE was observed (data not shown). From these experiments, it is not clear whether the addition of DMEM results in increased induction of the ICE, or increased permissiveness of the recipient.

Palmieri recently reported a 15 kb mobile genetic element harbouring antibiotic resistance genes that could exist either independently in episomal form, or integrated into a larger ICE, ICE*Su*32457 [23]. The 15 kb region could also be lost from transconjugants after transfer. The genes involved in excision, conjugation and integration in ICE*Sde*3396 and ICE*Su*32457 are almost identical. The major difference between the two ICEs is the 15 kb region and Region 2 which both carry accessory genes. Unlike ICE*Su*32457 all transconjugants examined in this study retained arsenate resistance (data not shown), indicating that firstly, arsenate resistance is not lost through gene mutation or deletion, at least within the scale of this study. Additionally, our results indicate that unlike ICE*Su*32457 and the 15 kb mobile genetic element, Region 2 is stably integrated into the chromosome.

## Conclusion

In summary we have shown that ICE*Sde*3396 is transmissible from multiple β-hemolytic species, and has the necessary attributes to be able to act as an efficient vehicle for dissemination of genes through the β-hemolytic streptococcal population. The presence of similar ICEs in *S. suis* [24], suggests this family of ICEs can also disseminate through a broader streptococcal population, thereby increasing the pool of genetic material that can be imported in by β-hemolytic streptococcal population. In particular the related ICEs in *S. suis* harbour antibiotic resistance genes. Acquisition of antibiotic resistance cassettes by ICE*Sde*3396, or alternatively, transfer of the ICEs from *S. suis* to β-hemolytic streptococci represent a potential mechanism by which antibiotic resistance may become fixed in specific strains in these populations.

## Methods

### Bacterial strains and molecular methods

Clinical and epidemiological details of the bacterial strains used in this study have been previously described [14, 15, 25, 26] and are summarised in Table 4. Streptococcal isolates were grown in Todd-Hewitt broth (THB), Todd Hewitt agar (THA) or Columbia Blood agar (CBA). SDSE NS3396 ICE*Sde*3396:km is a recombinant strain [15] that harbours ICE*Sde*3396 marked with a kanamycin resistance gene in Region 2 (Figure 1). Streptococci were rendered spontaneously resistant to streptomycin (str) or spectinomycin (sp) by growth in the presence of increasing concentrations of these antibiotics. SDSE and GBS are also intrinsically resistant to bacitracin (bc). The concentrations of antibiotics used in the study were as follows: kanamycin (500 μg/ml), streptomycin (400 μg/ml), spectinomycin (1 mg/ml) and bacitracin (0.8 μg/ml). Use of recombinantly altered streptococci, and transfer of ICESde3396:km to recipient strains was granted by the Office of Gene Technology Regulator of the Australian Government.

**Table 4:** **Bacterial strains used in this study**

Strain	Emm type/serotype	Relevant features ^a^	Source or reference
***S. dysgalactiae*** **subsp.** ***equisimilis*** **(SDSE)**			
NS3396	stg480	bc^r^, ICE*Sde*3396	[25]
NS3396:km	stg480	bc^r^, ICE*Sde*3396:km	[15]
GGS10str	stg62647	bc^r^, str^r^	This study
GGS10str/km	stg62647	bc^r^, str^r^, ICE*Sde*3396:km	This study
GGS10	stg62647	bc^r^	[25]
GGS10bc/km	stg62647	bc^r^, ICE*Sde*3396:km	This study
G120	stg4831	bc^r^	[15]
MD128	stg93464	bc^r^	[15]
NS1121	stg4831	bc^r^	[15]
NS383	New type	bc^r^	[15]
***S.pyogenes (*** **GAS** ***)***			
NS235str	emm24	str^r^	This study
NS235str/km	emm24	str^r^, ICE*Sde*3396:km	This study
NS235sp	emm24	sp^r^	This study
NS235sp/km	emm24	sp^r^, ICE*Sde*3396:km	This study
NS1185	N.D		[26]
NS344	emm1		[26]
NS20	emm75.1		[26]
NS351	emm58		[26]
NS672	N.D		[26]
***S. agalactiae (*** **GBS** ***)***			
RBH05str	V	bc^r^, str^r^	This study
RBH05str/km RBH05	V	bc^r^, str^r^, ICE*Sde*3396:km bc^r^	[15]
RBH05bc/km	V	bc^r^, ICE*Sde*3396:km	This study
B36PS	IV	bc^r^	This study
RBH04	Ia/V	bc^r^	[15]
RBH06	II	bc^r^	[15]
RBH08	Ia	bc^r^	[15]
RBH09	V	bc^r^	[15]
RBH10	V	bc^r^	[15]
RBH11	III	bc^r^	[15]
RBH14	Ib	bc^r^	[15]

^a^bc, bacitracin; km, kanamycin; str, streptomycin; sp, spectinomycin. N.D: not determined.

Chromosomal DNA was extracted using the RBC HiYield Genomic DNA Mini kit, modified by the addition of 10 U mutanolysin (Sigma) and 0.5 mg proteinase K (Promega) in the lysis steps. Primers and conditions for amplification of discrete regions of Region 1 and Region 3 have been previously described [15]. In this study Region 2 was amplified using R1P forward (5′-atagtttggcagcgaggaaa-3′) and reverse (5′-cgcatgacttcccattcagc-3′) primers. Amplification of DNA spanning the integration site of ICE*Sde*3396 in GBS chromosomal DNA was achieved with ICE66f (5′-tttgccattcgacctctttc-3′) and rplLr (5′-gtgaaatcacaggcgaaggt-3′) under standard conditions. Southern hybridisations were performed using standard protocols. Briefly, 2 μg of *Nde*I restricted chromosomal DNA was electrophoresed in 0.7% agarose gel in 1×TAE buffer and transferred to nitrocellulose membrane (Hybond-N, Amersham). The blots were incubated with digoxigenin-UTP labelled probes, and hybridisation detected using anti-DIG Alkaline Phosphatase conjugated antibody (Roche Diagnostics) and CDP-*Star* detection reagent (Tropix). The R3P probe used for hybdrisation here was amplified using forward (R3Pf, 5′-ggctcctactgccaatcaagc-3′) and reverse (R3Pr, 5′-gattgcggtcacaacagcta-3′) primers.

### Conjugation

Unless otherwise stated, conjugation experiments were performed using a microfuge method developed for streptococcal conjugation in this study. Five hundred microliters of overnight cultures of donor and recipient (~1 × 10^8^ CFU/ml) were mixed in 1.5 ml microcentrifuge tubes. Controls were prepared by mixing 500 μl of the donor or recipient culture mixed with 500 μl of THB. The suspensions were centrifuged at 3000 *g*, supernatant removed and pellet resuspended in 1 ml of THB. The samples were centrifuged again to facilitate contact between bacteria. After incubation at 37°C for between 10 min and 3 hours the samples were centrifuged, washed, plated onto THA or CBA containing kanamycin, selective for ICE*Sde*3396:km, and a second antibiotic, selective for the recipient strain. The plates were incubated overnight at 37°C and colony forming units (CFU) determined. Serially diluted samples were also plated onto agar containing a single antibiotic to score for the recovery of donors and recipients. Conjugation efficiency was calculated by dividing the number of transconjugants by the number of donors recovered per unit volume.

### Conjugation in the presence of eukaryotic cells

Eukaryotic A549 epithelial cells were grown to confluence in six-well plates in Dulbecco’s Modified Eagle Medium (DMEM) supplemented with 10% Fetal Calf Serum (FCS) at 37°C under 5% CO_2_. The wells were then inoculated with donors (~10^6^ CFU) and recipients (~10^7^ CFU). For control experiments streptococci were added to fresh DMEM or cell culture supernatant (spent medium). The plates were incubated for six hours at 37°C under 5% CO_2_. Post incubation, the supernatant was transferred to a new microfuge tube. To collect bacteria that might have adhered to the eukaryotic cell surface each well was also treated with 0.25% trypsin and 0.0125% Triton X-100 and detached cells added to the above tubes. The resulting suspension was centrifuged, resuspended in PBS and plated onto agar containing appropriate antibiotics.
